# Onset of schizophrenia diagnoses in a large clinical cohort

**DOI:** 10.1038/s41598-019-46109-8

**Published:** 2019-07-08

**Authors:** Jorge Lopez-Castroman, José Miguel Leiva-Murillo, Fanny Cegla-Schvartzman, Hilario Blasco-Fontecilla, Rebeca Garcia-Nieto, Antonio Artes-Rodriguez, Consuelo Morant-Ginestar, Philippe Courtet, Carlos Blanco, Fuensanta Aroca, Enrique Baca-García

**Affiliations:** 1 INSERM Unit 1061 and University of Montpellier, Montpellier, France; 2 Nimes University Hospital, Nimes, France; 3 Department of Signal Theory and Communications, Telecommunication Engineering Faculty, Carlos III University, Edif. Torres Quevedo, Avda. Universidad 30, 28911 Leganes, Madrid Spain; 4 Department of Psychiatry Hospital Universitario Fundación Jiménez Díaz, Madrid, Spain; 5 Department of Psychiatry, IDIPHIPSA-Puerta de Hierro University Hospital, CIBERSAM, UAM, Madrid, Spain; 6 Department of Mental Health, Madrid Regional Health Council, Madrid, Spain; 7 Department of Emergency Psychiatry and Post-Acute Care, CHRU Montpellier F-34000, Montpellier, France; 8 Department of Psychiatry at the New York State Psychiatric Institute and Columbia University, New York, USA; 9 Instituto de Matemáticas, UNAM, Mexico city, Mexico; 10 Department of Psychiatry, Universidad Autónoma, Madrid, Spain; 11 Department of Psychiatry, Hospital Universitario Rey Juan Carlos, Móstoles, Spain; 12 Department of Psychiatry, Hospital General de Villalba, Madrid, Spain; 13 Department of Psychiatry, Hospital Universitario Infanta Elena, Valdemoro, Spain; 14 CIBERSAM (Centro de Investigación en Salud Mental), Carlos III Institute of Health, Madrid, Spain; 15 Universidad Católica del Maule, Talca, Chile

**Keywords:** Health services, Epidemiology, Risk factors

## Abstract

We aimed to describe the diagnostic patterns preceding and following the onset of schizophrenia diagnoses in outpatient clinics. A large clinical sample of 26,163 patients with a diagnosis of schizophrenia in at least one outpatient visit was investigated. We applied a Continuous Time Hidden Markov Model to describe the probability of transition from other diagnoses to schizophrenia considering time proximity. Although the most frequent diagnoses before schizophrenia were anxiety and mood disorders, direct transitions to schizophrenia usually came from psychotic-spectrum disorders. The initial diagnosis of schizophrenia was not likely to change for two of every three patients if it was confirmed some months after its onset. When not confirmed, the most frequent alternative diagnoses were personality, affective or non-schizophrenia psychotic disorders. Misdiagnosis or comorbidity with affective, anxiety and personality disorders are frequent before and after the diagnosis of schizophrenia. Our findings give partial support to a dimensional view of schizophrenia and emphasize the need for longitudinal assessment.

## Introduction

Schizophrenia and schizoaffective disorder have a lifetime prevalence of about 1% and are among the leading causes of disability^1–5^. Due to its early onset and its deteriorating course, schizophrenia causes an immense economic burden. A large majority of patients with schizophrenia are unemployed, and impairments in functioning across social, vocational and residential domains remain severe even during periods of remission from active psychosis^6–10^, resulting in costs estimated at $62 billion in the US in 2002 and increasing three times its value a decade later. The economic burden of schizophrenia is estimated at $155.7 billion for 2013 including excess direct health care costs of $37.7 billion^11,12^. Approximately 80% of patients with schizophrenia relapse within 5 years of the first episode and many do not fully recover^13^. Moreover, the cognitive deficits and lack of insight that are core features of schizophrenia impair the patients’ ability to recognize their disability or the symptoms that precede a relapse^14^. The disorder is therefore a permanent source of anguish for patients and their families and is associated with an increased risk for suicide and general medical conditions^3,15^. Although significant advances have been made in the understanding of the illness during the last 130 years (since Kraepelin’s original classification in 1887) the underpinnings of its etiology remain unknown. There are no biomarkers that could be regularly used in clinical practice for the diagnosis of schizophrenia^16,17^. Schizophrenia is also a very heterogeneous illness, as originally described by Bleuler in 1911 and later confirmed in several studies^18,19^. Thus, longitudinal validation provides one of the most direct evidences of diagnostic validity^20^.

Several studies have investigated the long-term diagnostic stability of schizophrenia. Meta-analytical evidence shows high prospective diagnostic stability in schizophrenia spectrum, but most of the literature was focused on small samples of first-episode psychosis or individuals at high-risk of schizophrenia^21–24^. There is also limited knowledge of the different diagnoses received by the patients around the onset of schizophrenia. Based on two epidemiological studies, An der Heiden *et al*. (2000) reported that the first psychotic episode in schizophrenia was preceded in 75% of patients by an average of 5 years of prodromic symptoms, usually negative and depressive symptoms^25,26^. Other authors have also noted the presence of prior behavioral and affective abnormalities in patients with schizophrenia^27,28^. Likewise, prodromal samples studied prospectively were frequently diagnosed with depression, anxiety or substance use disorders, particularly cannabis, before making a transition to psychosis^29–31^.

On the other hand, the current scientific paradigm is challenging the long-standing categorical perspective^32^, in favor of a more dimensional conceptualization of psychoses^33^. According to the dimensional model, an extended phenotype of schizophrenia in the general population (vulnerability) would underlie the less common clinical phenotype of schizophrenia^34^. High levels of severity in different symptom dimensions would lead to clinical assessment, identification of correlated symptoms in other dimensions and finally, the diagnosis of schizophrenia. We might expect prior diagnoses corresponding to the different dimensions that have been proposed (negative, affective, psychotic, and cognitive) when studying a large sample of patients with schizophrenia.

## Aims of The Study

To examine the diagnostic evolution of patients with schizophrenia before and after this diagnosis is made for the first time in public mental health facilities. Therefore, we investigated a large clinical sample to identify which diagnoses preceded and followed that of schizophrenia in those patients who remained in treatment. We hypothesized that the early onset of symptoms in different dimensions would be reflected in correspondingly different diagnostic pathways leading to the diagnosis of schizophrenia.

## Method

### Sample

The Madrid Psychiatric Registration System established in 1980 includes all individuals treated in public mental health centers in Madrid (Spain) until 2008. Public mental health centers are part of the National Health Services and provide free medical coverage to a catchment area of about 6,000,000 inhabitants and are funded through taxes. The Madrid Case Registry (*Registro Acumulativo de Casos de la Comunidad de Madrid*) is a naturalistic study of diagnostic stability and consistency over time of the mental disorders in the area. It includes information from all psychiatric visits to public outpatient mental health clinics in the province of Madrid, Spain between 1980 and 2008. The database includes sociodemographic information and clinical diagnoses. From 1980 to 1992, all diagnoses in the registry were coded according to the 9^th^ Revision of the International Classification of Diseases (ICD-9). Since 1992, diagnoses have been assigned according to the 10th Revision of the ICD (ICD-10). ICD-9 codes were converted to ICD-10 codes using the guidelines published by the World Health Organization (WHO, 1993). The treating clinician (a psychiatrist or clinical psychologist) entered the diagnostic codes at every follow-up visit. A maximum of 2 diagnoses per patient per visit were recorded. Diagnostic counts in this study include comorbidities (for instance, a F1-F3 diagnosis would count both as F1 and F3). A detailed description of the database can be obtained elsewhere^35^. For this study we selected patients who met the following inclusion criteria: 1) diagnosed with schizophrenia (ICD-10 category F20) during at least one visit and 2) at least three registered diagnoses by psychiatrists or clinical psychologists (in three different visits to the outpatient clinics). In the resulting subset of patients (n = 26,163) with a diagnosis of schizophrenia during at least one outpatient visit; we examined the diagnoses given to these patients during previous visits to public mental health clinics (i.e., prior to the diagnosis of schizophrenia). All methods were performed in accordance with the relevant guidelines and regulations. The Institutional Review Board of “Hospital 12 de Octubre” and “Fundación Jiménez Diaz” approved this study.

### Data analysis

The probability of maintaining or changing the diagnosis of schizophrenia in the outpatient mental health visits was calculated in the 48 months following the initial diagnosis of schizophrenia. Probabilities were computed considering all the assessments made in one-month time lapses after the initial diagnosis. For each month the total number of diagnoses was added and then divided proportionally according to their distribution. If a diagnosis was missing (due to a longer delay between visits), the diagnosis recorded in the previous month was used instead.

### Continuous-time hidden Markov model of longitudinal diagnostic shifts

In order to build a graph describing the sequence of diagnoses over time, a statistical model is needed that incorporates: (i) the frequencies of the transitions among mental disorders, (ii) the time lags between consecutive psychiatric visits, and (iii) the diagnostic uncertainty due to missing information in some of the records. We used a novel technique based on a continuous-time hidden Markov model (CT-HMM) to build the graphical model^36^. This method is based on a Hidden Markov model (HMM), which posits that starting from the current state of a stochastic system or process, it is possible to establish a description of its future probability, assuming that the measures are performed regularly in time. Nevertheless, in clinical practice that is not the case due to irregular or missed visits. Taking into account these cases, CT-HMM incorporates that both changes between hidden states and the appearance of new features can occur at any time^37^.

The model makes use of the following assumptions: (i) the different patients are instances of the same stochastic process to be modeled; (ii) the process is stationary, so that the intensity of the interaction between two diseases does not depend on the age of the subject; (iii) a patient stays in the same state (disease) until the time instant of the following medical claim; (iv) the Markov property holds, meaning that the future clinical history of a patient only depends on his/her present state, and is independent of the past. In this model, each element q_ij_ in the matrix of parameters describes the strength of the relation between diseases *i* and *j*^38^. The time lags between clinical events are also considered, and are represented as the sum of each row’s elements q_i_ = Σ_j_q_ij_, which is high if the average transition time between *i* and the next clinical event is short. Finally, records with incomplete or lost diagnoses are treated probabilistically as uncertain observations under some underlying “hidden” disorder. This uncertainty was reflected by the b_i(k)_ parameters, which described the probability of a certain diagnosis *k* if the real underlying diagnosis was *i*.

## Results

### Sample description

About half of the patients in the sample were male (n = 13,941; 53.3%). Mean age at the first assessment was 37.6 years (SD = 15.5) and mean age at the first diagnosis of schizophrenia was 39.3 years (SD = 14.9). At the time of their first visit to the mental health centers the patients were generally single (n = 15,741; 62.5%), and half of them were living with their family of origin (50.5%). The patients generally had low educational attainment: 46.0% had completed less than fifth grade (n = 10,997). Only one out of every four patients was working (n = 5,964; 26.5%) while most other patients were unemployed (n = 4,820; 21.5%), homemakers (n = 3,587; 16.0%), disabled (n = 2712; 12.1%), or studying (n = 2325; 10.4%). The following variables presented missing data over 2%: marital status (n = 965), educational level (n = 2,277), and working status (n = 3,737).

Patients with schizophrenia diagnoses made 1,455,063 visits to mental health centers (mean ± SD = 96.6 ± 174.7). The total number of visits with non-schizophrenia diagnoses prior to schizophrenia was 279,245, with an average of 18.03 visits per patient (SD = 28.81).

### Previous diagnoses

In the sample, 56.7% of individuals (14,883/26,163) had received another diagnosis prior to being diagnosed with schizophrenia. Table 1 describes the diagnoses received in the first outpatient assessment at the mental health centers. Table 2 details the most frequent diagnoses on a per-visit basis prior to the diagnosis of schizophrenia.

**Table 1:** Most frequent diagnoses at initial assessment.

ICD-10 diagnoses	Frequency	Percentage
F20 Schizophrenia	11330	43.3
F4 Neurotic, stress-related and somatoform disorders	4516	17.3
F3 Mood disorders	3402	13.0
F22 Persistent delusional disorders	1068	4.1
F6 Disorders of adult personality and behavior	1034	3.9
F1 Mental and behavioral disorders due to psychoactive substance use	981	3.7
F23 Acute and transient psychotic disorders	776	3.0
F29 Unspecified nonorganic psychosis	656	2.5
F9 Behavioral and emotional disorders with onset usually occurring in childhood and adolescence	345	1.3
F25 Schizoaffective disorders	276	1.1
F0 Organic mental disorders	263	1.0
F7 Mental retardation	211	0.8
F5 Behavioral syndromes associated with physiological disturbances and physical factors	205	0.8
F8 Disorders of psychological development	145	0.6
Total	24465	96.3

Only diagnoses made at least in 100 visits are listed.

**Table 2:** Most frequent diagnoses until schizophrenia.

ICD-10 diagnoses	Frequency	Percentage
F3 Mood disorders	77461	27.7
F4 Neurotic, stress-related and somatoform disorders	50468	18.1
F6 Disorders of adult personality and behavior	24257	8.7
F22 Persistent delusional disorders	17660	6.3
F23 Acute and transient psychotic disorders	11907	4.9
F1 Mental and behavioral disorders due to psychoactive substance use	11639	4.3
F29 Unspecified nonorganic psychosis	8632	4.2
F25 Schizoaffective disorders	7488	3.1
F0 Organic mental disorders	4661	2.7
F9 Behavioral and emotional disorders with onset usually occurring in childhood and adolescence	4563	1.7
F7 Mental retardation	3468	1.6
F3-F6	3147	1.2
F3-F4	2789	1.1
F5 Behavioral syndromes associated with physiological disturbances and physical factors	2762	1.0
F4-F6	2647	1.0
F8 Disorders of psychological development	1886	0.9
F1-F6	1528	0.7
F1-F3	1423	0.5
Total	252127	90.3

Only diagnoses made at least in 1000 visits are listed.

When comorbidities were included, the most frequent diagnostic categories prior to schizophrenia followed the order of diagnoses reported in Table 2: ‘mood disorders’ (F3; 31.3%), ‘neurotic, stress-related and somatoform disorders’ (F4; 21.2%), non-schizophrenia diagnoses within the category of ‘schizophrenia, schizotypal and delusional disorders’ (F2; 19.2%), and ‘disorders of adult personality and behavior’ (F6; 12.3%). With respect to psychotic disorders specifically, 7.2% of patients were previously diagnosed of persistent delusional disorders (F22), 5.0% of acute and transient psychotic disorders (F23), 3.7% of unspecified nonorganic psychosis (F29), and 3.1% of schizoaffective disorders (F25).

### Transition to schizophrenia

**Figure 1:**
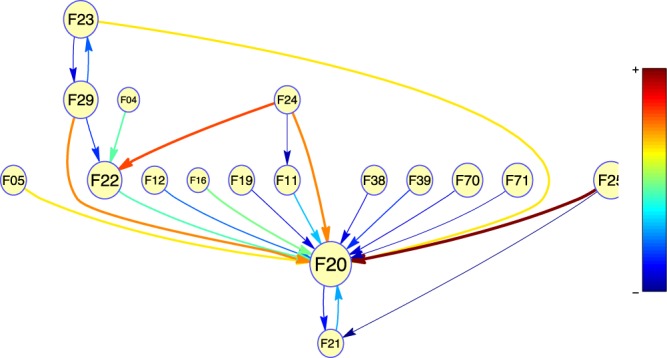
Probabilistic links of ICD-10 diagnoses converging into schizophrenia (F20). The size of the circles indicates the frequency of the diagnoses in our sample. The color and width of the arrows describe the strength of the interactions according to the model. F04/F05: Organic amnesic syndrome/Delirium, not induced by alcohol and other psychoactive substances; F11/12/F16/F19: Mental and behavioral disorders due to use of opioids/cannabinoids/hallucinogens/multiple drug use and use of other psychoactive substances; F21: Schizotypal disorder; F22: Persistent delusional disorders; F23: Acute and transient psychotic disorders; F24: Induced delusional disorder; F25: Schizoaffective disorders; F29: Unspecified nonorganic psychosis; F38: Other mood disorders; F39: Unspecified mood disorder; F70/F71: Mild/moderate mental retardation.

We then examined the probability of progression of these diagnoses to schizophrenia, considering also time proximity (Fig. 1). As might be expected, the strongest associations were between the psychotic-spectrum diagnoses and schizophrenia. From the highest to the lowest probability, schizoaffective (F25) disorders, induced delusional disorders (F24), unspecified nonorganic psychosis (F29), acute and transient psychotic disorders (F23), persistent delusional disorders (F22), and schizotypal disorders (F21) were all connected with a subsequent diagnosis of schizophrenia. The transitions to schizophrenia were indirect in some cases, usually through other psychotic disorders (for instance, F24 to F22 to F20). A probabilistic link from unspecified or other affective disorders (F38 and F39) towards schizophrenia was also represented, but not from bipolar disorder or major depression. Patients with alcohol, cannabis and multiple drug use disorders (F11, F12 and F19) were consequently diagnosed schizophrenia, but a direct transition was particularly frequent for the fewer subjects with hallucinogen use disorders (F16). Some organic mental disorders appeared also in our model. The diagnosis of delirium not induced by psychoactive substances (F05) showed a high probability of direct transition into schizophrenia, while the less frequent organic amnesic syndrome (F04) usually progressed to persistent delusional disorders before. Mild and moderate cases of mental retardation (F70 and F71) were also directly linked to schizophrenia in our model. Of note, several diagnostic categories, such as anxiety (F4) and personality disorders (F6), were not represented in the graphical model.

### Evolution/stability

**Figure 2:**
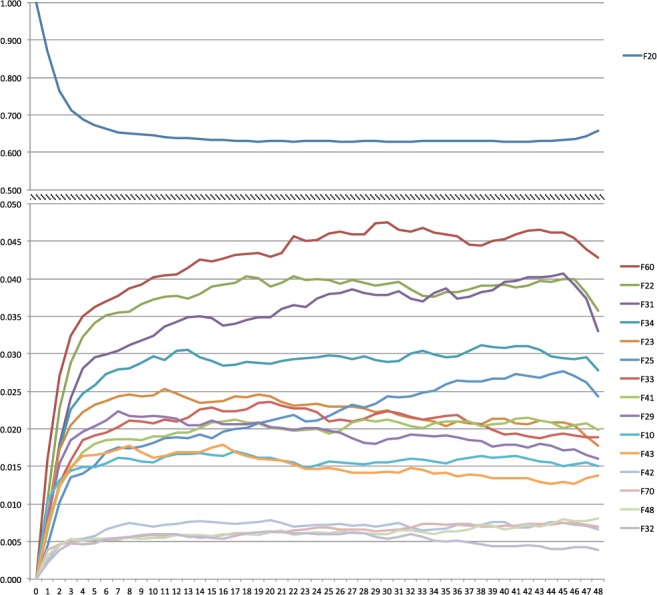
Diagnostic evolution of the first diagnosis of schizophrenia (F20) in the following 48 months. The upper section shows the probability (0.5–1) of maintaining the F20 diagnosis. The lower section shows the probability (0–0.05) of changing this diagnosis.

Diagnostic shift from schizophrenia was more commonly toward the following diagnoses, represented by the average percentage in the first 48 months: personality disorders (F60: 4.2%), delusional disorders (F22: 3.7%), bipolar disorder (F31: 3.5%), persistent mood disorders (F34: 2.8%), acute and transient psychotic disorders (F23: 2.2%) or schizoaffective disorder (F25: 2.1%). However, the majority (64.5%) of the patients with an initial diagnosis of schizophrenia continued to receive the same diagnosis in subsequent assessments (Fig. 2). Patients who had a diagnostic shift from schizophrenia to a non-schizophrenia diagnosis did so generally in the first six months after the diagnosis of schizophrenia had been made. After that time interval the rates of each diagnostic category remained stable.

## Discussion

This is a naturalistic study that describes which diagnoses the patients received before and after being diagnosed with schizophrenia for the first time. We found that although the most frequent prior diagnoses for those patients were anxiety (F4) and mood disorders (F3), direct transitions to schizophrenia usually came from psychotic-spectrum disorders. Furthermore, we also found that the initial diagnosis of schizophrenia is less likely to change if it is confirmed some months after its onset.

Indeed, psychotic symptoms have been noted as the best predictor of progression to schizophrenia among individuals at high risk^28^. Schizoaffective disorders are boundary diagnoses placed halfway between bipolar disorder and schizophrenia^16^. Schizotypal disorders and non-specified psychotic disorders are frequently used as a proxy for schizophrenia^23,33,39^. Therefore, it is not surprising that these diagnoses showed the most probable transitions to schizophrenia (Fig. 1). The uncommon diagnosis of induced delusional disorder also led to schizophrenia in the short term according to our model. The literature shows that a second diagnostic look sometimes reveals that a patient with supposedly induced delusional disorder shares the same genetically driven form of paranoid schizophrenia as an affected relative^40^. However, there are only a few case reports of this clinical entity^41^, and more studies are needed.

In line with the high prevalence of prodromal depressive symptomatology^29^, many patients with schizophrenia were previously diagnosed with anxiety or mood disorders. However, direct progression from affective or anxiety disorders did not appear as a probable transition to schizophrenia, implying that most patients received other diagnoses before eventually receiving a diagnosis of schizophrenia. In fact, anxiety and affective symptoms could be reactive expressions to prodromal and psychotic symptoms^42^. Personality disorders were the third most common diagnoses after mood and anxiety disorders, and likewise they were not directly followed by schizophrenia. This might reflect the uncertainty of the clinicians, who try to avoid direct transitions between conflicting diagnoses and perhaps some caution when it comes to diagnose a chronic and severe disorder such as schizophrenia. In other words, the high frequency of less severe previous diagnoses could be due to a conservative approach dealing with diagnostic uncertainty in early stages of a mental illness.

The direct transition between alcohol or drug use disorders represented in our model agrees with the mounting evidence of a causal influence of psychoactive substances in schizophrenia, particularly cannabis^43^. On the other hand, one study found that externalizing disorders were more frequent in the childhood and adolescence of patients with schizophrenia compared with those with bipolar disorder or depression^44^. However, behavioral and emotional disorders with onset usually occurring in childhood and adolescence (F9) were rarely registered before schizophrenia (1.3%) and the transition from F9 to schizophrenia was uncommon in our sample.

We also found that most patients retained the diagnosis of schizophrenia in the four years after its onset (65%). Although schizophrenia has a high diagnostic stability, according to previous works, prospective consistencies can vary from 52–100%^45–49^. In our sample approximately 35% of the patients diagnosed with schizophrenia, presented a change in their diagnosis (prospective consistency of 65%). This finding is consistent with the 70% temporal consistency that we have previously reported for schizophrenia in another sample, mostly assessed in outpatient facilities^48^. Overall, the results suggest that clinical assessment, which appears to be the most accurate diagnostic procedure for psychotic disorders^50^, repeatedly maintains the diagnosis of schizophrenia once it is confirmed. Moreover, the time lapse for its confirmation agrees with the 6-month current diagnostic criteria in DSM-IV, which has been described as excessively restrictive as it might lead to the detection of chronic subjects with a worse prognosis^51–53^. When not confirmed, the most frequent alternative diagnoses are personality, affective or non-schizophrenia psychotic disorders.

Even though the diagnosis of schizophrenia is more common in adolescence and early adulthood^54,55^, the risk of developing psychosis persists, and the age-incidence relationship seems to be altered by gender^56^. The evidence to date shows that men have an increased incidence at the end of the second decade and the beginning of the third, subsequently presenting lower rates, which remain thereafter. However, in women the first peak presents later on, with a smaller second peak in middle age^55^ and at 65 years, possibly a third peak^57^. In our cohort the observed age of onset fits on the upper limit reported in the literature (20–40 years at disease onset^58^). More than 50% of Kraepelin patients had onset of symptoms between ages 30 to 40, and over 20% between ages 40 to 50^59^.

Novel approaches, supported by recent epidemiological and clinical research^60,61^, try to consider the differential weight of dimensional traits of schizophrenia. In this study we expected to find a diagnostic pattern before schizophrenia that would correspond to the four symptom dimensions described in dimensional models^34^. Prior affective disorders and non-schizophrenia psychotic disorders were frequent in our sample, although the former seem to precede the latter in most cases. The other two symptom dimensions proposed in patients with schizophrenia (negative and cognitive) were rarely translated into specific diagnoses. This might suggest either that clinicians disregarded negative and cognitive symptoms as being part of a more severe clinical picture (affective or psychotic) or that an asymmetrical model of dimensions involved in schizophrenia would fit epidemiological data more closely. It should be noted that diagnostic classifications have evolved over time. The diagnostic criteria of ICD 9 are based on Schneider´s first rank symptoms and overlook negative and cognitive symptoms. This is less true for ICD 10 criteria.

This study examines clinical practice in Spain in real-world conditions, as it evaluates the follow-up of a large sample of patients with schizophrenia consecutively recruited in a 30-year interval. The representativeness of the study is enhanced by the free access and wide coverage of public medical care in our country. Moreover, since residential changes to other provinces are infrequent in Spain (<2% per year)^62^ and visits to other mental health centers of the region would be included in the registry, our data is likely to reflect the real-world diagnostic pathways of the patients. However, some patients had probably received the first diagnosis of schizophrenia before being included in the registry or outside the system (e.g., in a private consultation). Thus, our results likely represent not only incidence cases but also prevalent cases that have increased the mean age at first diagnosis. However, other studies have also reported similar mean age at first diagnosis in schizophrenia. Despite this weakness, our results still provide an estimate of the shift patterns in the diagnosis of schizophrenia among patients who were retained in the public mental health system of Madrid. On the other hand, as structured diagnostic instruments were not used, it is unclear to what extent the clinical picture is changing immediately prior to the diagnosis of schizophrenia or in the early phases of illness, or whether clinicians vary in their ability to recognize the disorder. However, clinical evaluation seems so far to be the best diagnostic tool for schizophrenia^50^, providing reliable and valid diagnoses when performed by mental health-specialists^63^.

The evolution of diagnoses before and after that of schizophrenia indicates frequent initial misdiagnoses or comorbidity with affective, anxiety and personality disorders. Nevertheless, a diagnosis of schizophrenia is usually reached from psychotic-spectrum disorders or directly assigned, and once reached it is confirmed in the following six months for two of every three patients.

## Data Availability

The datasets used and/or analyzed during the current study are available from the corresponding author on reasonable request.

## References

[1] Lehman AF (2004). Practice guideline for the treatment of patients with schizophrenia, second edition. Am J Psychiatry.

[2] Harvey PD (2012). Functional milestones and clinician ratings of everyday functioning in people with schizophrenia: overlap between milestones and specificity of ratings. J Psychiatr Res.

[3] Rossler W, Salize HJ, van Os J, Riecher-Rossler A (2005). Size of burden of schizophrenia and psychotic disorders. Eur Neuropsychopharmacol.

[4] Janoutova J (2016). Epidemiology and risk factors of schizophrenia. Neuro Endocrinol Lett.

[5] Murray, C. & Lopez, A. World Health Organization, World Bank & Harvard School of Public Health. The global burden of disease. *Harvard School of Public Health* (1996).

[6] Smith K (2011). Trillion-dollar brain drain. Nature.

[7] Chen R (2018). Assessment of functioning and disability in patients with schizophrenia using the WHO Disability Assessment Schedule 2.0 in a large-scale database. Eur Arch Psychiatry Clin Neurosci.

[8] Sabbag S (2012). Predictors of the accuracy of self assessment of everyday functioning in people with schizophrenia. Schizophr Res.

[9] Lundin, L. & Flyckt, L. [Schizophrenia past and present–the perception of long term prognoses have changed]. *Lakartidningen* **112** (2015). 26461504

[10] Harrow M (2004). Followup of psychotic outpatients: dimensions of delusions and work functioning in schizophrenia. Schizophr Bull.

[11] Wu EQ (2005). The economic burden of schizophrenia in the United States in 2002. J Clin Psychiatry.

[12] Cloutier M (2016). The Economic Burden of Schizophrenia in the United States in 2013. J Clin Psychiatry.

[13] Palmier-Claus JE (2012). The feasibility and validity of ambulatory self-report of psychotic symptoms using a smartphone software application. BMC Psychiatry.

[14] Insel TR (2010). Rethinking schizophrenia. Nature.

[15] Jobe TH, Harrow M (2005). Long-term outcome of patients with schizophrenia: a review. Can J Psychiatry.

[16] Nasrallah H, Tandon R, Keshavan M (2011). Beyond the facts in schizophrenia: closing the gaps in diagnosis, pathophysiology, and treatment. Epidemiol Psychiatr Sci.

[17] Birnbaum R, Weinberger DR (2017). Genetic insights into the neurodevelopmental origins of schizophrenia. Nat Rev Neurosci.

[18] Liddle PF (1987). The symptoms of chronic schizophrenia. A re-examination of the positive-negative dichotomy. Br J Psychiatry Aug.

[19] Schroder J (1996). Cerebral metabolic activity correlates of subsyndromes in chronic schizophrenia. Schizophrn Res. Mar.

[20] Robins E, Guze SB (1970). Establishment of diagnostic validity in psychiatric illness: its application to schizophrenia. American Journal of Psychiatry.

[21] Chang WC, Pang SL, Chung DW, Chan SS (2009). Five-year stability of ICD-10 diagnoses among Chinese patients presented with first-episode psychosis in Hong Kong. Schizophr Res.

[22] Bromet EJ (2011). Diagnostic shifts during the decade following first admission for psychosis. Am J Psychiatry.

[23] Salvatore P (2011). McLean-Harvard International First-Episode Project: two-year stability of ICD-10 diagnoses in 500 first-episode psychotic disorder patients. J Clin Psychiatry.

[24] Fusar-Poli P (2016). Diagnostic Stability of ICD/DSM First Episode Psychosis Diagnoses: Meta-analysis. Schizophr Bull.

[25] der Heiden W, Hafner H (2000). The epidemiology of onset and course of schizophrenia. Eur Arch Psychiatry Clin Neurosci.

[26] Hafner H, Maurer K, Trendler G, an der Heiden W, Schmidt M (2005). The early course of schizophrenia and depression*. Eur Arch Psychiatry Clin Neurosci.

[27] Moller P, Husby R (2000). The initial prodrome in schizophrenia: searching for naturalistic core dimensions of experience and behavior. Schizophr Bull.

[28] Miller PM, Lawrie SM, Byrne M, Cosway R, Johnstone EC (2002). Self-rated schizotypal cognitions, psychotic symptoms and the onset of schizophrenia in young people at high risk of schizophrenia. Acta Psychiatr Scand.

[29] Addington J (2011). At clinical high risk for psychosis: outcome for nonconverters. Am J Psychiatry.

[30] Rosen JL, Miller TJ, D’Andrea JT, McGlashan TH, Woods SW (2006). Comorbid diagnoses in patients meeting criteria for the schizophrenia prodrome. Schizophr Res.

[31] Sara GE, Burgess PM, Malhi GS, Whiteford HA, Hall WC (2014). The impact of cannabis and stimulant disorders on diagnostic stability in psychosis. J Clin Psychiatry.

[32] Murray RM, Dutta R (2007). The right answer for the wrong reasons?. World Psychiatry.

[33] Carpenter WT (2009). The psychoses: cluster 3 of the proposed meta-structure for DSM-V and ICD-11. Psychol Med.

[34] van Os J, Kenis G, Rutten BP (2010). The environment and schizophrenia. Nature.

[35] Carballo, J. J. *et al*. Continuity of Depressive Disorders From Childhood and Adolescence to Adulthood: A Naturalistic Study in Community Mental Health Centers. *Prim Care Companion CNS Disord* **13** (2011). 10.4088/PCC.11m01150 PMC3267511 22295270

[36] Leiva, J., Artes, A. & Baca, E. In *NIPS Workshop on Personalized Medicine* (Granada (Spain), 2011).

[37] Liu Y, Li S, Li F, Song L, Rehg JM (2015). Efficient Learning of Continuous-Time Hidden Markov Models for Disease Progression. Adv Neural Inf Process Syst.

[38] Kijima, M. *Markov Processes for Stochastic Modeling*. (Chapman & Hall, 1997).

[39] Owens DG, Johnstone EC (2006). Precursors and prodromata of schizophrenia: findings from the Edinburgh High Risk Study and their literature context. Psychol Med.

[40] Reif A, Pfuhlmann B (2004). Folie a deux versus genetically driven delusional disorder: case reports and nosological considerations. Compr Psychiatry.

[41] Mouchet-Mages S, Gourevitch R, Loo H (2008). [Folie a deux: update of an old concept regarding two cases]. Encephale.

[42] Yung AR (2007). Association between psychotic experiences and depression in a clinical sample over 6 months. Schizophr Res.

[43] Macleod J (2004). Psychological and social sequelae of cannabis and other illicit drug use by young people: a systematic review of longitudinal, general population studies. Lancet.

[44] Rubino IA (2009). A comparative study of axis I antecedents before age 18 of unipolar depression, bipolar disorder and schizophrenia. Psychopathology.

[45] Munk-Jorgensen P (1985). The schizophrenia diagnosis in Denmark. A register-based investigation. Acta Psychiatr Scand.

[46] Forrester A, Owens DG, Johnstone EC (2001). Diagnostic stability in subjects with multiple admissions for psychotic illness. Psychol Med.

[47] Amini H (2005). Diagnostic stability in patients with first-episode psychosis. Australas Psychiatry.

[48] Baca-Garcia E (2007). Diagnostic stability of psychiatric disorders in clinical practice. Br J Psychiatry.

[49] Atwoli L, Ndambuki D, Owiti P, Manguro G, Omulimi N (2012). Short-term diagnostic stability among re-admitted psychiatric in-patients in Eldoret, Kenya. Afr J Psychiatry (Johannesbg).

[50] Perala J (2007). Lifetime Prevalence of Psychotic and Bipolar I Disorders in a General Population. Archives General Psychiatry.

[51] Schwartz JE (2000). Congruence of diagnoses 2 years after a first-admission diagnosis of psychosis. Archives General Psychiatry.

[52] Maj M (1998). Critique of the DSM-IV operational diagnostic criteria for schizophrenia. Br J Psychiatry.

[53] Veen N (2004). Diagnostic stability in a Dutch psychosis incidence cohort. British Journal of Psychiatry.

[54] Weinberger DR (1987). Implications of normal brain development for the pathogenesis of schizophrenia. Arch Gen Psychiatry.

[55] Hafner H, Maurer K, Loffler W, Riecher-Rossler A (1993). The influence of age and sex on the onset and early course of schizophrenia. Br J Psychiatry.

[56] Angermeyer MC, Kuhn L (1988). Gender differences in age at onset of schizophrenia. An overview. Eur Arch Psychiatry Neurol Sci.

[57] Hafner H (1989). How does gender influence age at first hospitalization for schizophrenia? A transnational case register study. Psychol Med.

[58] Chen L, Selvendra A, Stewart A, Castle D (2018). Risk factors in early and late onset schizophrenia. Compr Psychiatry.

[59] Harris MJ, Jeste DV (1988). Late-onset schizophrenia: an overview. Schizophr Bull.

[60] Wiles NJ (2006). Self-reported psychotic symptoms in the general population: results from the longitudinal study of the British National Psychiatric Morbidity Survey. Br J Psychiatry.

[61] Esterberg ML, Compton MT (2009). The psychosis continuum and categorical versus dimensional diagnostic approaches. Curr Psychiatry Rep.

[62] Instituto Nacional de Estadistica. *Estadística de variaciones residenciales*. *Serie 1998–2011*, www.ine.es (2012).

[63] Harvey PD (2012). Diagnosis of schizophrenia: consistency across information sources and stability of the condition. Schizophr Res.

